# Association of Maternal Psychosocial Stress With Increased Risk of Asthma Development in Offspring

**DOI:** 10.1093/aje/kwx366

**Published:** 2017-12-13

**Authors:** Maria C Magnus, Rosalind J Wright, Espen Røysamb, Christine L Parr, Øystein Karlstad, Christian M Page, Per Nafstad, Siri E Håberg, Stephanie J London, Wenche Nystad

**Affiliations:** 1 Division of Mental and Physical Health, Norwegian Institute of Public Health, Oslo, Norway; 2 MRC Integrative Epidemiology Unit, University of Bristol, Bristol, United Kingdom; 3 Department of Population Health Sciences, Bristol Medical School, University of Bristol, Bristol, United Kingdom; 4 Department of Pediatrics, Icahn School of Medicine at Mount Sinai, New York, New York; 5 Department of Psychology, Faculty of Social Sciences, University of Oslo, Oslo, Norway; 6 Oslo Centre for Biostatistics and Epidemiology, Oslo University Hospital, Oslo, Norway; 7 Department of Community Medicine, Medical Faculty, University of Oslo, Oslo, Norway; 8 Epidemiology Branch, National Institute of Environmental Health Sciences, Research Triangle Park, North Carolina

**Keywords:** asthma, maternal stress, paternal stress, pregnancy, psychosocial stress

## Abstract

Prenatal maternal psychosocial stress might influence the development of childhood asthma. Evaluating paternal psychosocial stress and conducting a sibling comparison could provide further insight into the role of unmeasured confounding. We examined the associations of parental psychosocial stress during and after pregnancy with asthma at age 7 years in the Norwegian Mother and Child Cohort Study (*n* = 63,626; children born in 2000–2007). Measures of psychosocial stress included lifetime major depressive symptoms, current anxiety/depression symptoms, use of antidepressants, anxiolytics, and/or hypnotics, life satisfaction, relationship satisfaction, work stress, and social support. Childhood asthma was associated with maternal lifetime major depressive symptoms (adjusted relative risk (aRR) = 1.19, 95% confidence interval (CI): 1.09, 1.30), in addition to symptoms of anxiety/depression during pregnancy (aRR = 1.17, 95% CI: 1.06, 1.29) and 6 months after delivery (aRR = 1.17, 95% CI: 1.07, 1.28). Maternal negative life events during pregnancy (aRR = 1.10, 95% CI: 1.06, 1.13) and 6 months after delivery (aRR = 1.14, 95% CI: 1.11, 1.18) were also associated with asthma. These associations were not replicated when evaluated within sibling groups. There were no associations with paternal psychosocial stress. In conclusion, maternal anxiety/depression and negative life events were associated with offspring asthma, but this might be explained by unmeasured maternal background characteristics that remain stable across deliveries.

Prenatal exposure to maternal psychosocial stress is associated with increased risk of childhood asthma (1–16). Previous studies mostly evaluated single measures or a limited number of measures of psychosocial stress or stress correlates (e.g., psychological functioning), including maternal negative life events (1, 4, 7, 11, 15), depression/anxiety (3, 6, 12, 14, 16), community violence (2), bereavement (5, 8, 13), job strain (9), and demoralization (10). These studies also varied in the outcome definition, where some evaluated wheezing symptoms (2, 10–12, 15) and others evaluated asthma (1, 3–9, 13, 14). Furthermore, most of these studies relied on maternal reporting of childhood asthma, while only a few used information from national registries/administrative databases (5, 8, 13, 14).

We wanted to further examine whether the previously reported associations between maternal psychosocial stress and its correlates (hereafter denoted psychosocial stress) and risk of childhood asthma might be explained by unmeasured confounding, by using paternal psychosocial stress as a negative control and by conducting a sibling analysis (17, 18). If similar associations of maternal and paternal psychosocial stress with childhood asthma were observed, this could indicate that the reported association between maternal psychosocial stress and childhood asthma is explained by unmeasured background characteristics linked to psychosocial stress in both parents. A few previous studies examined paternal anxiety/depression while the mother was pregnant in relation to childhood asthma, reporting contradictory findings (6, 14). To the best of our knowledge, no previous study of parental psychosocial stress and childhood asthma used a sibling comparison in order to evaluate the potential role of unmeasured confounding by maternal background characteristics that remain stable between deliveries.

Our aim was therefore to estimate the associations of maternal psychosocial stress during and after pregnancy with childhood asthma. We also evaluated associations with paternal psychosocial stress and explored a sibling analysis to evaluate the role of unmeasured confounding. Because this study was performed in Norway, a country with universal access to health-care services and relatively low levels of social inequality, potential confounding by socioeconomic factors was less likely in comparison with many other locations.

## METHODS

### Study population

**Figure 1.**
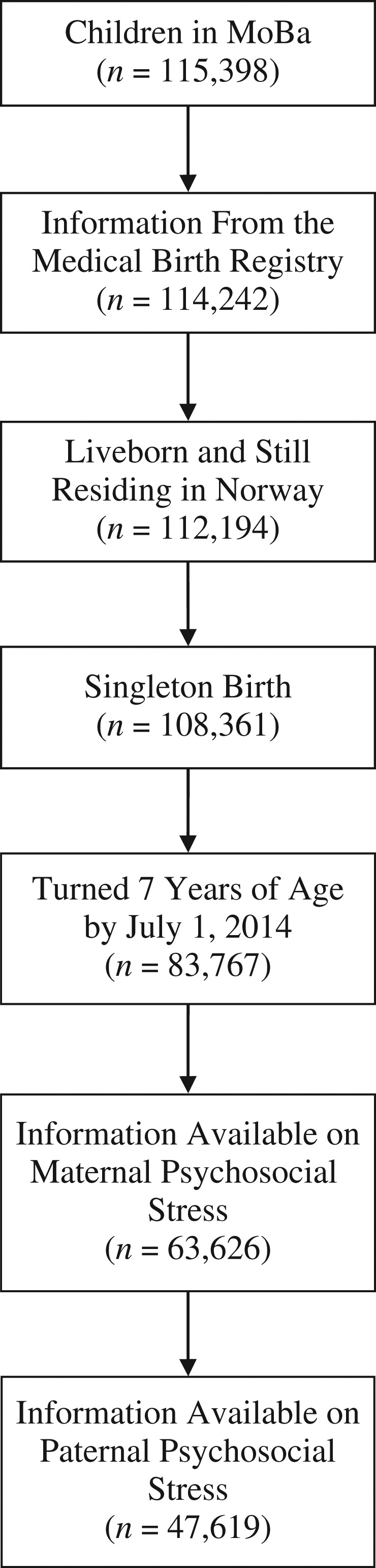
Selection of participants for a study of the association between maternal psychosocial stress and risk of asthma development in offspring, Norwegian Mother and Child Cohort Study (MoBa), 2000–2007.

We used data from the Norwegian Mother and Child Cohort Study (MoBa) (19, 20), which recruited pregnant women from all over Norway between 1999 and 2008, at approximately 18 weeks’ gestation. All participants in MoBa gave written informed consent, and the participation rate was 41%. Mothers could participate with more than 1 pregnancy, which resulted in the inclusion of approximately 95,200 mothers and 114,500 children. Information collected through MoBa questionnaires was subsequently linked to the Medical Birth Registry of Norway and the Norwegian Prescription Database, using national identification numbers. Dispensed prescriptions in the Norwegian Prescription Database are coded according to the Anatomical Therapeutic Chemical (ATC) classification system (21). A total of 83,767 liveborn singletons born before July 1, 2007, who had reached age 7 years by July 1, 2014, were eligible for the current study. Among these children, 63,626 had information on maternal psychosocial stress gathered at 18 weeks’ gestation, at 30 weeks’ gestation, and 6 months after delivery (Figure 1) and were included in the analysis of maternal psychosocial stress. In comparison, 47,619 children were available for the analysis of paternal psychosocial stress (Figure 1). This study was approved by the Norwegian Data Inspectorate and the Regional Committee for Medical and Health Research Ethics of South/East Norway.

### Maternal psychosocial stress during pregnancy

The specific questions asked to capture information on psychosocial stress are displayed in Web Table 1 (available at https://academic.oup.com/aje). We defined maternal lifetime history of major depressive symptoms based on at least 3 affirmative answers to a previously validated 5-item scale (22). Current self-reported symptoms of anxiety/depression were measured using a 5-item symptom checklist (SCL-5) at both 18 and 30 weeks of gestation (23). The answers were obtained on a 4-point Likert scale (Cronbach’s α = 0.80 and 0.81 at weeks 18 and 30, respectively), and we used the mean value to indicate the overall burden of symptoms. Mothers also reported their use of antidepressants, anxiolytics, and hypnotics during pregnancy at 18 and 30 weeks’ gestation, and retrospectively when the child was 6 months of age. Specific medications included antidepressants (ATC codes N06AB, N06AF, N06AG, and N06AX), benzodiazepines and benzodiazepine-related drugs (ATC codes N05BA, N05CD, N03AE01, and N05CF), buspiron (ATC code N05BE01), pregabalin (ATC code N03AX16), and the hypnotic drug melatonin (ATC code N05CH01).

We also measured maternal self-reported life satisfaction with a 5-item scale at both 18 and 30 weeks’ gestation (24–26), where overall life satisfaction was defined as the mean of the responses gathered on a 7-point Likert scale and low scores represented dissatisfaction (Cronbach’s α = 0.89 at both 18 and 30 weeks). Mothers also responded to 10 questions related to relationship satisfaction at both 18 and 30 weeks’ gestation (27). The answers were gathered on a 6-point Likert scale, where the scale was reversed for negatively phrased questions, and the mean value across the 10 items was used as a measure of overall relationship satisfaction (Cronbach’s α = 0.91 at both 18 and 30 weeks). Participants also reported whether they had experienced any of 8 different negative life events during the past year at 30 weeks’ gestation (28, 29), and the answers were added to indicate the total burden of negative life events. Work stress was reported at 18 weeks’ gestation using 8 questions, with answers on a 4-point Likert scale. After reversing the answers to positively phrased questions, the mean value was used as an overall measure of work stress, with higher values indicating higher levels of work stress. Finally, we defined social support as the mean of the answers to 3 questions at 18 weeks’ gestation.

### Paternal psychosocial stress during pregnancy and maternal psychosocial stress after pregnancy

We also obtained information on paternal self-reported lifetime history of major depressive symptoms, current symptoms of anxiety/depression (SCL-5 score; continuous), use of antidepressants, anxiolytics, and/or hypnotics, self-reported satisfaction with life, and relationship satisfaction at the time of recruitment. Mothers further reported current symptoms of anxiety/depression (SCL-5 score), use of antidepressants, anxiolytics, and/or hypnotics, satisfaction with life, and relationship satisfaction, in addition to negative life events occurring after 30 weeks’ gestation, when the child was aged 6 months.

### Childhood asthma

We defined asthma at age 7 years on the basis of dispensed asthma medication during the past year, in addition to a second dispensed prescription within 1 year after the first, as registered in the Norwegian Prescription Database. Asthma medications included long-acting β_2_-agonists (ATC code R03AC), inhaled corticosteroids (ATC code R03BA), fixed-dose combinations of inhaled β_2_-agonists and corticosteroids (ATC code R03AK), and/or leukotriene antagonists (ATC code R03DC). We also assessed maternal reporting of current asthma on the 7-year questionnaire as a secondary outcome, defined as a maternal report of physician-diagnosed asthma, in combination with either current symptoms or use of asthma medication in the past year.

### Covariates

We obtained additional information on characteristics that could plausibly be associated with psychosocial stress and childhood asthma. Maternal characteristics included age at delivery, parity (0, 1, 2, or ≥3), education (less than high school, high school, ≤4 years of college, or >4 years of college), prepregnancy body mass index (weight (kg)/height (m)^2^; <18.5, 18.5–24.9, 25–29.9, or ≥30), smoking during pregnancy (none, quit by 18 weeks, or smoked after 18 weeks), smoking during the first 6 months of the child’s life (none, occasionally, or daily), and history of asthma. Paternal characteristics included age at delivery (<25, 25–29, 30–34, or ≥35 years), education (less than high school, high school, ≤4 years of college, or >4 years of college)), body mass index (<18.5, 18.5–24.9, 25–29.9, or ≥30), smoking (yes vs. no), and history of asthma. Finally, child characteristics included sex, birth weight in grams, gestational age in weeks, and whether the child was still being breastfed at 6 months of age (yes vs. no).

### Statistical analysis

We evaluated parental psychosocial stress in relation to childhood asthma using log-binomial regression, reporting relative risks and 95% confidence intervals. In order to account for the presence of siblings, we used robust cluster variance estimation. We adjusted results of the analysis of maternal psychosocial stress during pregnancy in relation to childhood asthma for maternal age, parity, education, prepregnancy body mass index, smoking during pregnancy, and history of asthma. We did not adjust the association between maternal psychosocial stress during pregnancy and childhood asthma for pregnancy complications, since they could lie on the causal pathway and we were interested in examining the total association. However, the associations with maternal psychosocial stress 6 months after delivery was further adjusted for smoking 6 months after delivery, child sex, birth weight, gestational age, and whether the child was still being breastfed at age 6 months. The associations with paternal psychosocial stress were adjusted for paternal age, education, smoking, body mass index, and history of asthma.

We conducted a number of sensitivity analyses. First, we adjusted results on maternal symptoms of anxiety/depression for the use of antidepressants, anxiolytics, and/or hypnotics during pregnancy. Second, we adjusted the associations with maternal psychosocial stress 6 months after delivery for the same measures of psychosocial stress during pregnancy. Third, we evaluated the associations with maternal prenatal psychosocial stress in the subsample with information on paternal psychosocial stress. Fourth, we explored mutual adjustment of the same measures of maternal and paternal psychosocial stress. To examine selection bias due to the restriction of the main analysis to persons with information from the questionnaires administered at 18 weeks’ gestation, at 30 weeks’ gestation, and 6 months after delivery, we reevaluated these analyses without restricting the results to persons with information from the questionnaire administered 6 months after delivery. We also conducted a secondary analysis of maternal report of asthma at age 7 years in the subgroup with information from the 7-year follow-up questionnaire (*n* = 37,694). Finally, we also conducted a sibling analysis using fixed-effects logistic regression, reporting odds ratios and 95% confidence intervals. The fixed-effects logistic regression compared the distributions of the exposure among sibling pairs discordant for the outcome of interest. The analysis was therefore restricted to discordant sibling pairs and was equivalent to a conditional logistic regression used for matched case-control studies, where the discordant sibling pairs are the matched pairs of interest.

Fewer than 5% of observations were excluded from any of the multivariable analyses because of missing information on 1 or more covariates. The results presented are therefore from a complete-case analysis. We conducted the analyses using Stata, version 14 (StataCorp LP, College Station, Texas).

## RESULTS

The distribution of background characteristics among children included in the analyses of maternal and paternal psychosocial stress is given in Table 1. When we compared included and excluded children on the basis of necessary follow-up information, the children included had parents who were older, more educated, and less likely to be smokers and more mothers who were nulliparous (Web Table 2). There was no appreciable difference in the proportions of children with asthma among participants with (4.2%) and without (4.5%) information on measures of maternal psychosocial stress (Web Table 2). Likewise, the proportions of children with asthma among children with (4.1%) and without (4.5%) information on measures of paternal psychosocial stress were very similar (Web Table 2).

**Table 1.** Distribution of Background Characteristics Among Children Included in an Analysis of Maternal and Paternal Stress and Childhood Asthma, Norwegian Mother and Child Cohort Study, 2000–2007

Background Characteristic	Maternal Psychosocial Stress (*n* = 63,626)		Paternal Psychosocial Stress (*n* = 47,619)	
	No. of Children	%	No. of Children	%
Maternal age at delivery, years^a^	30.1 (4.5)		30.1 (4.4)	
Maternal parity				
0	28,413	44.7	22,374	47.0
1	22,650	35.6	16,617	34.9
2	9,849	15.5	6,860	14.4
≥3	2,714	4.3	1,768	3.7
Maternal education				
Less than high school	4,795	7.5	3,274	6.9
High school	19,318	30.4	13,851	29.1
≤4 years of college	26,261	41.3	20,021	42.0
>4 years of college	12,996	20.4	10,267	21.6
Missing data	256	0.4	206	0.4
Maternal prepregnancy body mass index^b^				
<18.5	1,817	2.9	1,340	2.8
18.5–24.9	40,453	63.6	30,259	63.5
25–29.9	13,854	21.8	10,397	21.8
≥30	5,858	9.2	4,435	9.3
Missing data	1,644	2.6	1,188	2.5
Maternal smoking during pregnancy				
None	48,184	75.7	36,654	77.0
Quit by 18 weeks	8,991	14.1	6,661	14.0
Smoked after 18 weeks	6,182	9.7	4,112	8.6
Missing data	269	0.4	192	0.4
Maternal smoking during first 6 months of child’s life				
No	52,124	81.9	39,517	83.0
Yes	8,879	14.0	6,174	13.0
Missing data	2,623	4.1	1,928	4.1
Maternal history of asthma				
No	58,924	92.6	44,027	92.5
Yes	4,702	7.4	3,592	7.5
Paternal age, years				
<25	2,851	4.5	2,083	4.4
25–29	14,592	22.9	11,040	23.2
30–34	25,101	39.5	19,235	40.4
≥35	20,955	32.9	15,259	32.0
Missing data	127	0.2	2	0
Paternal education				
Less than high school	6,772	10.6	4,441	9.3
High school	25,631	40.3	18,691	39.3
≤4 years of college	16,679	26.2	13,082	27.5
>4 years of college	12,638	19.9	10,089	21.2
Missing data	1,906	3.0	1,316	2.8
Paternal body mass index				
<18.5	128	0.2	99	0.2
18.5–24.9	27,149	42.7	20,122	42.3
25–29.9	27,565	43.3	20,846	43.8
≥30	5,829	9.2	4,560	9.6
Missing data	2,955	4.6	1,992	4.2
Paternal smoking				
No	45,830	72.0	35,388	74.3
Yes	17,490	27.5	12,050	25.3
Missing data	306	0.5	181	0.4
Paternal history of asthma				
No	43,462	68.3	43,462	91.3
Yes	4,157	6.5	4,157	8.7
Missing data	16,007	25.2	0	0
Child’s sex				
Male	32,562	51.2	24,329	51.1
Female	31,064	48.8	23,290	48.9
Child’s birth weight, g^a^	3,621 (545)		3,613 (543)	
Missing data	31	0.1	22	0.1
Child’s gestational age, weeks^a^	39.5 (1.7)		39.5 (1.7)	
Missing data	244	0.4	188	0.4
Child still breastfeeding at age 6 months				
No	11,554	18.2	8,574	18.0
Yes	52,072	81.8	39,045	82.0

^a^ Values are expressed as mean (standard deviation).

^b^ Weight (kg)/height (m)^2^.

There was a modest correlation between the different measures of maternal psychosocial stress during pregnancy, ranging from 0.11 to 0.78 (Web Table 3). Maternal lifetime history of major depressive symptoms (adjusted relative risk (aRR) = 1.19, 95% confidence interval (CI): 1.09, 1.30), in addition to symptoms of anxiety/depression during pregnancy (SCL-5) (per unit increase in the symptom score, aRR = 1.19, 95% CI: 1.09, 1.30), was positively associated with asthma at age 7 years (Table 2). There was no strong evidence for an association with maternal use of antidepressants, anxiolytics, and/or hypnotics during pregnancy (Table 2). Additional adjustment of the association between maternal symptoms of anxiety/depression during pregnancy and childhood asthma for the use of antidepressants, anxiolytics, and/or hypnotics did not change the associations (Web Table 4). When we evaluated maternal use of antidepressants, anxiolytics, and hypnotics during pregnancy separately, we found no strong evidence for an association between any of these medication groups and childhood asthma (Web Table 5). Maternal negative life events during pregnancy were also positively associated with asthma at age 7 years (per additional negative life event, aRR = 1.10, 95% CI: 1.06, 1.13) (Table 2). There was no strong evidence of associations with maternal satisfaction with life, relationship satisfaction, or work stress (Table 2). The sensitivity analysis reexamining the associations with maternal psychosocial stress at 18 and 30 weeks’ gestation without restricting the data to persons with information from the questionnaire administered 6 months after delivery yielded similar findings (Web Table 6).

**Table 2.** Association Between Maternal Psychosocial Stress During Pregnancy and Offspring Asthma at Age 7 Years as Defined by the Norwegian Prescription Database, Norwegian Mother and Child Cohort Study, 2000–2007

Pregnancy Period and Measure of Maternal Psychosocial Stress	Range of Scale Scores	No. of Children	No. of Cases	Median (IQR)	Unadjusted		Adjusted^a^	
					RR	95% CI	RR	95% CI
18 weeks’ gestation								
SCL-5 score^b^	1–4	62,438	2,610	1.0 (1.0–1.4)	1.31	1.20, 1.42	1.19	1.09, 1.30
Satisfaction With Life Scale^c^	1–7	62,877	2,629	5.8 (5.2–6.4)	0.94	0.91, 0.98	0.99	0.95, 1.03
Relationship satisfaction^c^	1–6	61,039	2,534	5.5 (5.0–5.8)	0.97	0.92, 1.03	1.01	0.96, 1.08
Work stress^b^	1–4	58,547	2,374	2.0 (1.8–2.3)	1.21	1.10, 1.33	1.08	0.97, 1.20
Social support^c^	1–3	61,742	2,575	3.3 (3.0–3.7)	0.86	0.79, 0.94	0.94	0.86, 1.03
Lifetime symptoms of depression^b^	0–5	62,922	2,630	2.0 (0.0–3.0)	1.08	1.06, 1.10	1.05	1.02, 1.07
Lifetime history of major depressive symptoms								
No		49,056	1,920	N/A	1.00	Referent	1.00	Referent
Yes		13,866	710	N/A	1.31	1.20, 1.42	1.19	1.09, 1.30
30 weeks’ gestation								
SCL-5 score^b^	1–4	63,112	2,631	1.1 (1.0–1.4)	1.30	1.18, 1.43	1.17	1.06, 1.29
Satisfaction With Life Scale^c^	1–7	63,158	2,636	6.0 (5.4–6.2)	0.98	0.94, 1.02	1.02	0.98, 1.07
Relationship satisfaction^c^	1–6	62,487	2,602	5.5 (5.1–5.8)	1.02	0.96, 1.09	1.06	0.99, 1.13
Negative life events during the past year^b^	0–8	63,626	2,659	1.0 (0.0–1.0)	1.15	1.11, 1.19	1.10	1.06, 1.13
Use of antidepressants, anxiolytics, and/or hypnotics during pregnancy								
No		62,560	2,602	N/A	1.00	Referent	1.00	Referent
Yes		1,066	57	N/A	1.29	1.00, 1.66	1.16	0.90, 1.50

Abbreviations: CI, confidence interval; IQR, interquartile range; N/A, not applicable; RR, relative risk; SCL-5, 5-item symptom checklist.

^a^ Adjusted for maternal age, parity, education, prepregnancy body mass index, smoking during pregnancy, and history of asthma.

^b^ Higher scores indicate more stress.

^c^ Higher scores indicate less stress.

We also evaluated associations of paternal psychosocial stress at the time of recruitment (during pregnancy) with asthma at age 7 years, and the correlation between the different measures of paternal psychosocial stress is shown in Web Table 7. The correlations between the same measures of parental psychosocial stress at the time of recruitment ranged from 0.18 to 0.55, with the strongest correlation being seen for relationship satisfaction (Web Table 8). We observed no strong evidence for associations of paternal psychosocial stress with asthma at age 7 years (Table 3). For example, the association between paternal symptoms of anxiety/depression and asthma yielded an adjusted relative risk of 1.05 (95% CI: 0.90, 1.23) per unit increase in the symptom score (Table 3). Examining the associations with maternal psychosocial stress during pregnancy in the subsample in the analysis of paternal psychosocial stress yielded similar associations with maternal psychosocial stress (Web Table 9). Mutual adjustment for the same measures of parental psychosocial stress during pregnancy also did not change the observed associations with maternal psychosocial stress during pregnancy (Web Table 10).

**Table 3.** Association Between Paternal Psychosocial Stress and Offspring Asthma at Age 7 Years as Defined by the Norwegian Prescription Database, Norwegian Mother and Child Cohort Study, 2000–2007

Measure of Paternal Psychosocial Stress	Range of Scale Scores	No. of Children	No. of Cases	Median (IQR)	Unadjusted		Adjusted^a^	
					RR	95% CI	RR	95% CI
SCL-5 score^b^	1–4	47,133	1,925	1.0 (1.0–1.2)	1.10	0.95, 1.27	1.05	0.90, 1.23
Satisfaction With Life Scale^c^	1–7	47,191	1,924	5.8 (5.2–6.2)	0.96	0.92, 1.01	0.97	0.93, 1.02
Relationship satisfaction^c^	1–6	47,033	1,917	5.4 (5.0–5.8)	1.03	0.95, 1.11	1.03	0.95, 1.12
Lifetime symptoms of depression^b^	0–5	47,088	1,925	0.0 (0.0–2.0)	1.05	1.02, 1.09	1.03	1.00, 1.07
Lifetime history of major depressive symptoms								
No		42,568	1,746	N/A	1.00	Referent	1.00	Referent
Yes		4,520	179	N/A	0.97	0.83, 1.12	0.95	0.81, 1.11
Use of antidepressants, anxiolytics, and/or hypnotics during pregnancy								
No		46,894	1,911	N/A	1.00	Referent	1.00	Referent
Yes		725	28	N/A	0.95	0.66, 1.37	0.81	0.55, 1.20

Abbreviations: CI, confidence interval; IQR, interquartile range; N/A, not applicable; RR, relative risk; SCL-5, 5-item symptom checklist.

^a^ Adjusted for paternal age, education, body mass index, smoking, and history of asthma.

^b^ Higher scores indicate more stress.

^c^ Higher scores indicate less stress.

The correlations between measures of maternal psychosocial stress during pregnancy and the same measures 6 months after delivery ranged from 0.56 to 0.78. Maternal symptoms of anxiety/depression reported 6 months after delivery (per unit increase in the symptom score, aRR = 1.17, 95% CI: 1.07, 1.28) and negative life events (per additional negative life event, aRR = 1.14, 95% CI: 1.11, 1.18) were positively associated with asthma at age 7 years (Table 4). The association between maternal symptoms of anxiety/depression reported 6 months after delivery and asthma at age 7 years was attenuated after adjustment for symptoms during pregnancy (Table 4). In contrast, the association between negative life events reported 6 months after delivery and asthma at age 7 years remained largely unchanged after adjustment for negative life events reported during pregnancy (Table 4).

**Table 4.** Association Between Maternal Psychosocial Stress 6 Months After Delivery and Offspring Asthma at Age 7 Years as Defined by the Norwegian Prescription Database, Norwegian Mother and Child Cohort Study, 2000–2007

Measure of Maternal Psychosocial Stress 6 Months After Delivery	Range of Scale Scores	No. of Children	No. of Cases	Median (IQR)	Unadjusted		Adjusted			
							Model 1^a^		Model 2^b^	
					RR	95% CI	RR	95% CI	RR	95% CI
SCL-5 score^c^	1–4	63,332	2,649	1.0 (1.0–1.4)	1.30	1.19, 1.41	1.17	1.07, 1.28	1.09	0.98, 1.21
Satisfaction With Life Scale^d^	1–7	62,844	2,626	6.0 (5.4–6.4)	0.92	0.89, 0.95	0.96	0.93, 1.00	0.96	0.91, 1.00
Relationship satisfaction^d^	1–6	61,679	2,553	5.4 (5.0–5.8)	0.96	0.91, 1.01	1.00	0.95, 1.06	1.00	0.93, 1.08
Negative life events after 30 weeks’ gestation^c^	0–10	63,626	2,659	0.0 (0.0–1.0)	1.20	1.17, 1.24	1.14	1.11, 1.18	1.12	1.09, 1.16
Use of antidepressants, anxiolytics, and/or hypnotics during pregnancy										
No		62,860	2,621	N/A	1.00	Referent	1.00	Referent	1.00	Referent
Yes		766	38	N/A	1.19	0.87, 1.63	1.01	0.73, 1.41	0.95	0.67, 1.34

Abbreviations: CI, confidence interval; IQR, interquartile range; N/A, not applicable; RR, relative risk; SCL-5, 5-item symptom checklist.

^a^ Model 1 adjusted for maternal age, parity, education, smoking during pregnancy, prepregnancy body mass index, smoking during the first 6 months of the child’s life, and history of asthma, in addition to child’s sex, birth weight, gestational age, and breastfeeding during the first 6 months of life.

^b^ Model 2 adjusted for the characteristics described for model 1 and for maternal report of the same measure of psychosocial stress during pregnancy.

^c^ Higher scores indicates more stress.

^d^ Higher scores indicates less stress.

The secondary analyses of parental psychosocial stress with asthma at age 7 years defined on the basis of maternal report through questionnaires yielded results similar to those of the main analysis (Web Tables 11–13). When we evaluated the associations within sibling groups, maternal symptoms of anxiety/depression before, during, or after pregnancy showed no evidence of an association with offspring asthma at age 7 years (Tables 5 and 6). However, the sibling analyses indicated a positive association between maternal relationship satisfaction and offspring asthma at age 7 years that was not observed in the primary/population overall analysis (Tables 5 and 6). The sibling analyses yielded similar associations with paternal psychosocial stress (Web Table 14).

**Table 5.** Results From a Sibling Analysis of Maternal Psychosocial Stress During Pregnancy and Offspring Asthma at Age 7 Years as Defined by the Norwegian Prescription Database, Norwegian Mother and Child Cohort Study, 2000–2007

Pregnancy Period and Measure of Maternal Psychosocial Stress	Range of Scale Scores	No. of Children	No. of Cases	Unadjusted		Adjusted^a^	
				OR	95% CI	OR	95% CI
18 weeks’ gestation							
SCL-5 score^b^	1–4	848	416	0.97	0.58, 1.61	1.00	0.56, 1.77
Satisfaction With Life Scale^c^	1–7	857	437	0.98	0.83, 1.17	1.00	0.84, 1.20
Relationship satisfaction^c^	1–6	781	381	1.19	0.82, 1.75	1.37	0.88, 2.14
Work stress^b^	1–4	725	354	0.72	0.42, 1.26	0.69	0.37, 1.29
Social support^c^	1–3	795	388	1.04	0.62, 1.74	0.88	0.50, 1.56
Lifetime symptoms of depression^b^	0–5	860	421	0.89	0.76, 1.03	0.89	0.75, 1.04
Lifetime history of major depressive symptoms							
No		656	323	1.00	Referent	1.00	Referent
Yes		204	98	0.88	0.56, 1.39	0.90	0.55, 1.50
30 weeks’ gestation							
SCL-5 score^b^	1–4	865	423	0.74	0.43, 1.27	0.74	0.42, 1.33
Satisfaction With Life Scale^c^	1–7	861	421	1.05	0.86, 1.27	1.03	0.83, 1.27
Relationship satisfaction^c^	1–6	853	417	1.74	1.23, 2.48	2.06	1.33, 3.19
Negative life events during the past year^b^	0–8	867	424	1.07	0.92, 1.25	1.05	0.89, 1.24
Use of antidepressants, anxiolytics, and/or hypnotics during pregnancy							
No		855	418	1.00	Referent	1.00	Referent
Yes		12	6	1.00	0.29, 3.45	1.02	0.26, 3.96

Abbreviations: CI, confidence interval; OR, odds ratio; SCL-5, 5-item symptom checklist.

^a^ Adjusted for maternal age, parity, education, prepregnancy body mass index, smoking during pregnancy, and history of asthma.

^b^ Higher scores indicate more stress.

^c^ Higher scores indicate less stress.

**Table 6.** Results From a Sibling Analysis of Maternal Psychosocial Stress 6 Months After Delivery and Offspring Asthma at Age 7 Years as Defined by the Norwegian Prescription Database, Norwegian Mother and Child Cohort Study, 2000–2007

Measure of Maternal Psychosocial Stress 6 Months After Delivery	Range of Scale Scores	No. of Children	No. of Cases	Unadjusted		Adjusted			
						Model 1^a^		Model 2^b^	
				OR	95% CI	OR	95% CI	OR	95% CI
SCL-5 score^c^	1–4	865	423	1.37	0.80, 2.34	1.52	0.79, 2.91	1.46	0.75, 2.84
Satisfaction With Life Scale^d^	1–7	846	414	1.15	0.89, 1.48	1.28	0.94, 1.74	1.32	0.96, 1.80
Relationship satisfaction^d^	1–6	840	411	1.39	1.01, 1.90	1.35	0.91, 2.00	1.30	0.86, 1.96
Negative life events after 30 weeks’ gestation^c^	0–10	867	424	1.07	0.91, 1.26	1.10	0.91, 1.32	1.09	0.91, 1.32
Use of antidepressants, anxiolytics, and/or hypnotics during pregnancy									
No		857	420	1.00	Referent	1.00	Referent	1.00	Referent
Yes		10	4	0.60	0.14, 2.51	0.82	0.15, 4.42	0.82	0.15, 4.50

Abbreviations: CI, confidence interval; OR, odds ratio; SCL-5, 5-item symptom checklist.

^a^ Model 1 adjusted for maternal age, parity, education, smoking during pregnancy, prepregnancy body mass index, smoking during the first 6 months of the child’s life, and history of asthma, in addition to child’s sex, birth weight, gestational age, and breastfeeding during the first 6 months of life.

^b^ Model 2 adjusted for the characteristics described for model 1 and for maternal report of the same measure of psychosocial stress during pregnancy.

^c^ Higher scores indicates more stress.

^d^ Higher scores indicates less stress.

## DISCUSSION

In this large-scale pregnancy cohort from a country with universal access to health care and relatively modest social inequality, maternal symptoms of anxiety/depression and negative life events, both during pregnancy and during the first 6 months after delivery, were positively associated with asthma in children at age 7 years. Our findings were not explained by unmeasured confounding by background characteristics linked to these measures of psychosocial stress in both parents, since we observed no associations with paternal anxiety/depression. However, we cannot exclude the possibility of unmeasured confounding by maternal background characteristics that remain stable between deliveries based on our sibling analysis. Other measures of maternal psychosocial stress, such as satisfaction with life, relationship satisfaction, or work stress, showed no strong evidence of associations with the risk of childhood asthma.

Strengths of this study include the range of measures of psychosocial stress, the inclusion of paternal psychosocial stress as a negative control, the inclusion of a sibling comparison, and the modest likelihood of confounding due to social inequality. Selection bias could have arisen if either participation in MoBa or loss to follow-up was jointly associated with parental psychosocial stress and the child’s risk of asthma. Very reassuringly, the prevalence of asthma medication use in the past year at age 7 years among MoBa participants was very similar to the national estimated prevalence in Norway (30), and the proportions of children with asthma were similar among those with and without information on parental psychosocial stress. This indicates that neither participation in the cohort nor loss to follow-up was associated with our outcome of interest. Relying on self-reports of parental psychosocial stress could have resulted in misclassification. Assuming that any misclassification of parental psychosocial stress was not differential by offspring asthma, this would have attenuated the observed associations. In contrast, if mothers with higher levels of psychosocial stress are more likely to obtain health-care services for respiratory symptoms in the child, which could result in a higher proportion of their offspring being diagnosed with asthma, there is a chance of an ascertainment bias that could have led us to overestimate the true association. We relied on dispensed asthma medications as registered in the prescription registry to define asthma. However, there was a high concordance between maternal reports that the child used asthma medication at age 7 years and dispensed asthma medications (31). We also did not adjust for multiple testing due to the correlation between the measures of parental psychosocial stress, indicating that the tests were not strictly independent, and we can therefore not fully exclude the possibility of chance findings. We also cannot exclude the possibility of unobserved confounding of the observed associations by factors we could not adjust for, such as whether participants lived in an urban environment versus a rural environment.

Finally, due to the modest number of discordant sibling groups, our sibling analysis had less statistical power than the much larger primary analysis, which was reflected in the wider confidence intervals for the estimates obtained from this analysis. The sibling analysis also was more prone to bias by unmeasured parental background characteristics that change between deliveries, in addition to misclassification (32). For example, if siblings differ to a greater extent with regard to distributions of potential confounders than with regard to the exposure of interest, a sibling analysis may be more biased than a standard analysis (32, 33).

Our main findings support previously reported positive associations between maternal depression/anxiety and offspring asthma (3, 6, 14). In a study of the Generation R cohort, Guxens et al. (6) found an association between maternal symptoms of depression/anxiety during pregnancy and maternal reports of wheezing symptoms in the child’s first 6 years of life. This association was seemingly independent of paternal anxiety/depression and maternal anxiety/depression after delivery (6). Results from the Avon Longitudinal Study of Parents and Children further showed that maternal symptoms of anxiety during pregnancy were positively associated with maternal reports of children’s asthma at age 7 years (3). Finally, a large registry-based Danish study showed a positive association between maternal depression, both before and during pregnancy, and childhood asthma defined by hospital discharge codes and dispensed prescriptions, while there was no association with maternal use of medications for anxiety/depression—very similar to our findings (14). Our findings also support previous studies indicating a positive association between maternal negative life events and childhood asthma (1, 4, 7, 11, 15), although the measures of negative life events varied greatly across studies. In contrast, our findings are not in line with a previous report that maternal work stress during pregnancy is positively associated with asthma (9).

Since we did not observe an association with paternal anxiety/depression, the association between maternal anxiety/depression and childhood asthma might not be explained by unmeasured characteristics linked to this measure of psychosocial stress in both parents (17). These results correspond to what was reported for paternal anxiety/depression from the Generation R cohort (6) but are in contrast to the findings from the Danish registry study (14). Seemingly negating the findings from the negative control analysis, the findings from our sibling analyses indicate that the associations of maternal symptoms of anxiety/depression and negative life events with offspring asthma could be explained by unmeasured maternal background characteristics that remain stable between deliveries. However, due to the modest number of discordant sibling groups available for the sibling analyses and the fact that sibling analyses can actually be more biased under certain scenarios (32), we remain cautious in the interpretation of these findings.

Potential biological mechanisms exist for an influence of maternal psychosocial stress during pregnancy on the development of offspring asthma. In humans, exposure to prenatal psychosocial stress is also associated with altered umbilical cord blood cytokine responses (34) and elevated immunoglobulin E levels (35–37), which might predispose the child to allergic airway disease. One might also speculate that maternal psychosocial stress during pregnancy could influence epigenetic changes in the developing offspring (38, 39) and/or result in shortening of telomeres (40, 41), with potential consequences for asthma in the offspring. Additional research is necessary to further explore these potential mechanisms. The fact that we only observed associations for maternal symptoms of anxiety/depression and negative life events, and not other measures of psychosocial stress (satisfaction with life, relationship satisfaction, and work stress), could indicate that these 2 measures were capturing something more severe.

In conclusion, maternal anxiety/depression and negative life events, both during pregnancy and in the first 6 months after delivery, were associated with increased risk of asthma in the offspring, but this might be explained by unmeasured maternal background characteristics that remain stable across deliveries. The relatively modest levels of other measures of parental psychosocial stress (satisfaction with life, relationship satisfaction, and work stress) were not associated with childhood asthma in this cohort.

## Supplementary Material

Web Material

## Abbreviations

aRR: adjusted relative risk
ATC: Anatomical Therapeutic Chemical
CI: confidence interval
MoBa: Norwegian Mother and Child Cohort Study
OR: odds ratio
SCL-5: 5-item symptom checklist
