# Supplementary material for: Association of Maternal Psychosocial Stress With Increased Risk of Asthma Development in Offspring
Source: Am J Epidemiol. 2017 Dec 13;187(6):1199–209. doi: 10.1093/aje/kwx366 (PMC5982733; doi:10.1093/aje/kwx366)
Supplement: Web Material [file kwx366magnuswebmaterialfinal.pdf]

Web Table 1. Questions used to obtain information of psychosocial stress, Norwegian Mother and Child Cohort Study, 2000–2007

Scale Capturing Psychosocial Stress	Questions	Answer Options
Life-time history of major depressive symptoms	Felt depressed, sad?	Yes versus no
	Had problems with appetite or eaten too much?	
	Been bothered by feeling weak or a lack of energy?	
	Really blamed yourself and felt worthless?	
	Had problems with concentration or had problems making decisions?	
Five-item Symptom Checklist (SCL-5) for anxiety/depression	Constantly frightened or anxious?	A 4-point Likert scale ranging from 1 (not at all bothered) to 4 (very much bothered)
	Nervous, inner turmoil?	
	Feeling of hopelessness with regard to the future?	
	Depressed, sad?	
	Frequently worried or uneasy?	
Satisfaction With Life Scale (SWLS)	My life is largely what I wanted it to be	A seven point Likert scale ranging from disagree completely (1) to agree completely (7)
	My life is very good	
	I am satisfied with my life	
	To date, I have achieved what is important for me in my life	
	If I could start all over, there is very little I would do differently	
Social support	Do you have anyone other than your husband/partner you can ask for advice in a difficult situation? (Answer options	1-No, 2-Yes, 1 or 2 people, and 3-Yes, more than 2 people
	How often do you meet or talk on the telephone with your family (other than your husband/partner and children) or close friends?	1-Once a month or less, 2-2-8 times a month, and 3-More than twice a week
	Do you feel lonely?	1-Almost never, 2-Infrequently, 3-Sometimes, 4-Usually, and 5-Almost always
10-item Relationship Satisfaction (RS) scale	My husband/partner and I have a close relationship	A six point Likert scale ranging from (1) strongly disagree to (6) strongly agree
	My partner and I have problems in our relationship	
	I am very happy in my relationship	
	My partner is usually understanding	
	I often think about ending our relationship	
	I am satisfied with my relationship with my partner	
	We often disagree about important decisions	
	I have been lucky in my choice of a partner	
	We agree about how children should be raised	
	I think my partner is satisfied with our relationship	
Negative life events	Have you had problems at work or where you study?	Yes versus no
	Have you had financial problems?	
	Have you been divorced, separated or ended your relationship with your partner?	
	Have you had problems or conflicts with your family, friends or neighbors?	
	Have you been seriously ill or injured?	
	Has anyone close to you been seriously ill or injured?	
	Have you been involved in a serious accident, fire or robbery?	
	Have you lost someone close to you?	
Work stress	I have physically heavy work	A 4-point Likert scale ranging from (1) agree to (4) disagree
	My work is very stressful	
	I learn a lot at work	
	My work is very monotonous	
	My work demands a lot of me	
	I am able to decide how my work is to be carried out	
	There is a good team spirit at my place of work	
	I enjoy my work	

Web Table 2. Distribution of background characteristics (*N* (%)) among included and excluded individuals, Norwegian Mother and Child Cohort Study, 2000–2007

Background Characteristic	Maternal Psychosocial Stress		Paternal Psychosocial Stress	
	**Included (*N* = 63,626)**	**Excluded (*N* = 20,141)**	**Included (*N* = 47,619)**	**Excluded (*N* = 16,007)**
Child asthma use of asthma medications at 7 years in the NorPD				
No	60,967 (95.8)	19,230 (95.5)	45,680 (95.9)	15,287 (95.5)
Yes	2,659 (4.2)	911 (4.5)	1,939 (4.1)	720 (4.5)
Maternal age at delivery [a]	30.1 (4.5)	29.5 (5.1)	30.1 (4.4)	30.2 (4.7)
Maternal parity				
0	28,413 (44.7)	7,382 (36.7)	22,374 (47.0)	6,039 (37.7)
1	22,650 (35.6)	7,776 (38.6)	16,617 (34.9)	6,033 (37.7)
2	9,849 (15.5)	3,693 (18.3)	6,860 (14.4)	2,989 (18.7)
3+	2,714 (4.3)	1,290 (6.4)	1,768 (3.7)	946 (5.9)
Maternal education				
Less than high school	4,795 (7.6)	1,903 (15.8)	3,274 (6.9)	1,521 (9.5)
High school	19,318 (30.5)	4,167 (34.6)	13,851 (29.2)	5,467 (34.3)
Up to four years of college	26,261 (41.4)	3,962 (32.9)	20,021 (42.2)	6,240 (39.1)
More than four years of college	12,996 (20.5)	2,022 (16.8)	10,267 (21.7)	2,729 (17.1)
Missing	256	8,087	206	50
Maternal prepregnancy body mass index [b]				
<18.5	1,817 (2.9)	496 (4.3)	1,340 (2.9)	477 (3.1)
18.5-24.9	40,453 (65.3)	7,315 (63.0)	30,259 (65.2)	10,194 (65.6)
25-29.9	13,854 (22.4)	2,537 (21.9)	10,397 (22.4)	3,457 (22.2)
30 or higher	5,858 (9.5)	1,264 (10.9)	4,435 (9.6)	1,423 (9.2)
Missing	1,644	8,529	1,188	456
Maternal smoking during pregnancy				
No	48,184 (76.2)	8,380 (67.7)	36,654 (77.3)	11,530 (72.4)
Quit by 18 weeks	8,991 (14.2)	1,941 (15.7)	6,661 (14.0)	2,330 (14.6)
Smoked after 18 weeks	6,182 (9.8)	2,058 (16.6)	4,112 (8.7)	2,070 (13.0)
Missing	269	7,762	192	77
Maternal history of asthma				
No	58,924 (92.6)	11,159 (92.6)	44,027 (92.5)	14,897 (93.1)
Yes	4,702 (7.4)	1,012 (7.4)	3,592 (7.5)	1,110 (6.9)
Missing	0	7,970	0	0
Paternal age				
<25	2,851 (4.5)	1,623 (8.1)	2,083 (4.4)	768 (4.8)
25-29	14,592 (22.9)	4,657 (23.2)	11,040 (23.2)	3,552 (22.4)
30-34	25,101 (39.5)	7,161 (35.7)	19,235 (40.4)	5,866 (36.9)
35 and higher	20,955 (33.0)	6,609 (33.0)	15,259 (32.1)	5,696 (35.9)
Missing	127	91	2	125

Paternal education				
Less than high school	6,772 (11.0)	1,749 (15.2)	4,441 (9.6)	2,331 (15.1)
High school	25,631 (41.5)	5,003 (43.5)	18,691 (40.4)	6,940 (45.0)
Up to four years of college	16,679 (27.0)	2,593 (22.6)	13,082 (28.3)	3,597 (23.3)
More than four years of college	12,638 (20.5)	2,146 (18.7)	10,089 (21.8)	2,549 (16.5)
Missing	1,906	8,650	1,316	590
Paternal body mass index				
<18.5	128 (0.2)	34 (0.3)	99 (0.2)	29 (0.2)
18.5-24.9	27,149 (44.8)	5,059 (44.8)	20,122 (44.1)	7,027 (46.7)
25-29.9	27,565 (45.4)	5,005 (44.4)	20,846 (45.7)	6,719 (44.7)
30 or higher	5,829 (9.6)	1,186 (10.5)	4,560 (10.0)	1,269 (8.4)
Missing	2,955	8,857	1,992	963
Paternal smoking				
No	45,830 (72.4)	8,363 (67.5)	35,388 (74.6)	10,442 (65.8)
Yes	17,490 (27.6)	4,033 (32.5)	12,050 (25.4)	5,440 (34.3)
Missing	306	7,745	181	125
Paternal history of asthma				
No	43,462 (91.3)	7,510 (90.9)	43,462 (91.3)	NA
Yes	4,157 (8.7)	751 (9.1)	4,157 (8.7)	NA
Missing	16,007	11,880	0	NA

[a] Values are expressed as mean (standard deviation).

[b] Weight (kg)/height (m)^2.

Web Table 3. The pairwise correlation coefficients between the different measures of maternal psychosocial stress during pregnancy, Norwegian Mother and Child Cohort Study, 2000–2007

Measure of Maternal Psychosocial Stress	Symptom Checklist 5 (SCL-5) at 18 gw[a]	Satisfaction With Life Scale at 18 gw[b]	Relationship Satisfaction at 18 gw[b]	Work Stress at 18 gw[a]	Social Support at 18 gw[b]	Lifetime Symptoms of Depression[a]	Symptom Checklist 5 (SCL-5) at 30 gw[a]	Satisfaction With Life Scale at 30 gw[b]	Relationship Satisfaction at 30 gw[b]	Negative Life Events in Past 12 Months at 30 gw[a]
Symptom Checklist 5 (SCL-5) at 18 gw[a]	1									
Satisfaction With Life Scale at 18 gw[b]	-0.39	1								
Relationship satisfaction at 18 gw [b]	-0.31	0.44	1							
Work stress at 18 gw [a]	0.19	-0.23	-0.11	1						
Social support at 18 gw [b]	-0.32	0.33	0.30	-0.18	1					
Lifetime symptoms of depression[a]	0.38	-0.27	-0.19	0.13	-0.24	1				
Symptom Checklist 5 (SCL-5) at 30 gw [a]	0.58	-0.30	-0.24	0.16	-0.28	0.37	1			
Satisfaction With Life Scale at 30 gw [b]	-0.35	0.56	0.43	-0.20	0.32	-0.25	-0.37	1		
Relationship satisfaction at 30 gw [b]	-0.26	0.38	0.78	-0.11	0.29	-0.17	-0.31	0.49	1	
Negative life events the past 12 months at 30 gw [a]	0.28	-0.20	-0.15	0.16	-0.16	0.27	0.28	-0.21	-0.16	1

Abbreviation: gw, gestational weeks.

Web Table 4. Association between maternal symptoms of anxiety/depression during pregnancy and offspring asthma at 7 years defined by the Norwegian Prescription Database, Norwegian Mother and Child Cohort Study, 2000–2007

**Symptom Checklist 5 (SCL-5)**[a]	**Range of Scale**	***N***	**No. of Cases**	**Median (IQR)**	**Unadjusted**		**Adjusted** [b]	
					**RR**	**95% CI**	**RR**	**95% CI**
18 gestational weeks	1-4	62,438	2,610	1.0 (1.0, 1.4)	1.31	1.20, 1.42	1.19	1.09, 1.30
30 gestational weeks	1-4	63,112	2,631	1.1 (1.0, 1.4)	1.30	1.18, 1.43	1.16	1.05, 1.28

CI, confidence interval; IQR, interquartile range; RR, relative risk.

[a] Higher scores indicate more stress.

[b] Adjusted for maternal age, parity, education, prepregnancy body mass index, smoking during pregnancy, history of asthma and use of medications for anxiety/depression during pregnancy.

Web Table 5. Associations of maternal use of anxiolytics, antidepressants and hypnotics during pregnancy with offspring asthma at 7 years defined by the Norwegian Prescription Database, Norwegian Mother and Child Cohort Study, 2000–2007

Medication Group	Range of Scale	*N*	No. of Cases	Unadjusted		Adjusted [a]	
				RR	95% CI	RR	95% CI
Anxiolytics	No	63,273	2,642	Referent		Referent	
	Yes	353	17	1.15	0.72, 1.84	1.04	0.65, 1.64
Antidepressants	No	63,009	2,621	Referent		Referent	
	Yes	617	38	1.48	1.09, 2.02	1.27	0.92, 1.74
Hypnotics	No	63,381	2,650	Referent		Referent	
	Yes	245	9	0.88	0.46, 1.67	0.85	0.45, 1.62

CI, confidence interval; RR, relative risk.

[a] Adjusted for maternal age, parity, education, prepregnancy body mass index, smoking during pregnancy, and history of asthma.

Web Table 6. Association of maternal psychosocial stress during pregnancy with offspring asthma at 7 years defined by the Norwegian Prescription Database, Norwegian Mother and Child Cohort Study, 2000–2007: sensitivity analysis without restricting to individuals with information from the questionnaire administered 6 months after delivery

**Measure of Maternal Psychosocial Stress During Pregnancy**	**Range of Scale**	***N***	**No. of Cases**	**Median (IQR)**	**Unadjusted**		**Adjusted [a]**	
					**RR**	**95% CI**	**RR**	**95% CI**
Symptom Checklist 5 (SCL-5) at 18 gestational weeks [b]	1-4	74,071	3,147	1.0 (1.0, 1.4)	1.26	1.17, 1.36	1.17	1.08, 1.26
Satisfaction With Life Scale at 18 gestational weeks [c]	1-7	74,703	3,180	5.8 (5.2, 6.4)	0.96	0.93, 0.99	1.00	0.97, 1.03
Relationship satisfaction at 18 gestational weeks [c]	1-6	71,833	3,026	5.4 (5.0, 5.8)	0.97	0.92, 1.02	1.00	0.95, 1.06
Work stress at 18 gestational weeks [b]	1-4	68,830	2,855	3.0 (2.8, 3.3)	0.84	0.77, 0.92	0.94	0.85, 1.03
Social support at 18 gestational weeks [c]	1-3	72,857	3,086	3.3 (3.0, 3.7)	0.89	0.83, 0.96	0.96	0.88, 1.04
Lifetime symptoms of depression at 18 gestational weeks [b]	0-5	74,762	3,182	2.0 (0.0, 3.0)	1.08	1.06, 1.11	1.06	1.03, 1.08
Lifetime history of major depressive symptoms at 18 gestational weeks	No	57,692	2,298	NA	Referent		Referent	
	Yes	17,070	884	NA	1.30	1.20, 1.40	1.20	1.11, 1.30
Symptom Checklist 5 (SCL-5) at 30 gestational weeks [b]	1-4	69,296	2,910	1.1 (1.0, 1.4)	1.27	1.16, 1.38	1.15	1.04¸ 1.26
Satisfaction With Life Scale at 30 gestational weeks [c]	1-7	69,341	2,911	6.0 (5.4, 6.2)	0.97	0.94, 1.01	1.02	0.98, 1.05
Relationship satisfaction at 30 gestational weeks [c]	1-6	68,552	2,866	5.5 (5.0, 5.8)	1.01	0.95, 1.07	1.04	0.98, 1.10
Negative life events the past 12 months at 30 gestational weeks [b]	0-8	69,934	2,939	1.0 (0.0, 2.0)	1.14	1.11, 1.18	1.10	1.06, 1.13

CI, confidence interval; IQR, interquartile range; RR, relative risk.

[a] Adjusted for maternal age, parity, education, prepregnancy body mass index, smoking during pregnancy and history of asthma.

[b] Higher scores indicate more stress.

[c] Higher scores indicate less stress.

Web Table 7. The pairwise correlation coefficients between the different measures of paternal psychosocial stress ascertained at recruitment, Norwegian Mother and Child Cohort Study, 2000–2007

Measure of Paternal Psychosocial Stress	Symptom Checklist 5 (SCL-5)	Satisfaction With Life Scale at 18 gw	Relationship Satisfaction at 18 gw	Lifetime Symptoms of Depression
Symptom Checklist 5 (SCL-5)	1			
Satisfaction With Life Scale	−0.36	1		
Relationship satisfaction	−0.26	0.41	1	
Lifetime symptoms of depression	0.41	−0.29	−0.18	1

Abbreviation: gw, gestational weeks.

Web Table 8. The pairwise correlation coefficients of maternal and paternal psychosocial stress at recruitment around 18 gestational weeks, Norwegian Mother and Child Cohort Study, 2000–2007

Measure of Parental Psychosocial Stress	Correlation Coefficient
Symptom Checklist 5 (SCL-5)	0.19
Satisfaction With Life Scale	0.25
Relationship satisfaction	0.55
Lifetime symptoms of depression	0.18

Web Table 9. Association between maternal psychosocial stress during pregnancy and offspring asthma at 7 years defined by the Norwegian Prescription Database, Norwegian Mother and Child Cohort Study, 2000–2007: sensitivity analysis among study sample used to evaluate paternal psychosocial stress

Measure of Maternal Psychosocial Stress During Pregnancy	Range of Scale	*N*	No. of Cases	Median (IQR)	Unadjusted		Adjusted [a]	
					RR	95% CI	RR	95% CI
Symptom Checklist 5 (SCL-5) at 18 gestational weeks [b]	1-4	46,813	1,907	1.0 (1.0, 1.4)	1.29	1.17, 1.43	1.18	1.06, 1.31
Satisfaction With Life Scale at 18 gestational weeks [c]	1-7	47,108	1,915	6.0 (5.4, 6.4)	0.96	0.92, 1.00	1.01	0.96, 1.05
Relationship Satisfaction at 18 gestational weeks [c]	1-6	47,173	1,914	5.5 (5.1, 5.8)	0.99	0.92, 1.07	1.03	0.95, 1.12
Work stress at 18 gestational weeks [b]	1-4	43,980	1,736	3.0 (2.8, 3.3)	1.17	1.05, 1.31	1.02	0.90, 1.15
Social support at 18 gestational weeks [c]	1-3	47,378	1,926	3.3 (3.0, 3.7)	0.87	0.79, 0.96	0.95	0.85, 1.05
Lifetime symptoms of depression at 18 gestational weeks [b]	0-5	47,113	1,917	2.0 (0.0, 3.0)	1.07	1.05, 1.10	1.05	1.02, 1.07
Lifetime history of major depressive symptoms at 18 gestational weeks	No	36,816	1,419	NA	Referent		Referent	
	Yes	10,297	498	NA	1.25	1.14, 1.39	1.14	1.03, 1.27
Symptom Checklist 5 (SCL-5) at 30 gestational weeks [b]	1-4	47,273	1,921	1.1 (1.0, 1.4)	1.24	1.10, 1.39	1.10	0.97, 1.24
Satisfaction With Life Scale at 30 gestational weeks [c]	1-7	47,296	1,922	6.0 (5.4, 6.4)	1.00	0.95, 1.05	1.05	1.00, 1.10
Relationship Satisfaction at 30 gestational weeks [c]	1-6	47,161	1,922	5.5 (5.1, 5.8)	1.05	0.97, 1.14	1.09	1.01, 1.19
Negative Life Events at 30 gestational weeks [b]	0-8	47,619	1,939	1.0 (0.0, 1.0)	1.15	1.11, 1.12	1.10	1.06, 1.15
Use of antidepressants, anxiolytics and/or hypnotics during pregnancy	No	46,836	1,895	NA	Referent		Referent	
	Yes	783	44	NA	1.39	1.04, 1.86	1.23	0.91, 1.65

CI, confidence interval; IQR, interquartile range; RR, relative risk.

[a] Adjusted for maternal age, parity, education, prepregnancy body mass index, smoking during pregnancy and history of asthma.

[b] Higher scores indicate more stress.

[c] Higher scores indicate less stress.

Web Table 10. Associations of maternal and paternal psychosocial stress with offspring asthma at 7 years defined by the Norwegian Prescription Database after mutual adjustment, Norwegian Mother and Child Cohort Study, 2000–2007

Measure of Maternal Psychosocial Stress During Pregnancy at 18 Gestational Weeks	Range of Scale	*N*	No. of Cases	Median (IQR)	Unadjusted		Adjusted [a]	
					RR	95% CI	RR	95% CI
Symptom Checklist 5 (SCL-5) [b]	1-4	46,813	1,907	1.0 (1.0, 1.4)	1.29	1.17, 1.43	1.18	1.06, 1.32
Satisfaction With Life Scale [c]	1-7	47,108	1,915	6.0 (5.4, 6.4)	0.96	0.92, 1.00	1.01	0.94, 1.03
Relationship satisfaction [c]	1-6	47,173	47,173	5.5 (5.1, 5.8)	0.99	0.92, 1.07	1.00	0.91, 1.09
Lifetime symptoms of depression [b]	0-5	47,113	1,917	2.0 (0.0, 3.0)	1.07	1.05, 1.10	1.04	1.01, 1.07
Lifetime history of major depressive symptoms	No	36,816	1,419	NA	Referent		Referent	
	Yes	10,297	498	NA	1.25	1.14, 1.39	1.14	1.03, 1.27
**Measures of maternal psychosocial stress during pregnancy at 30 gestational weeks**								
Symptom Checklist 5 (SCL5) [b]	1-4	47,273	1,921	1.1 (1.0, 1.4)	1.24	1.10, 1.39	1.10	0.97, 1.24
Satisfaction With Life Scale [c]	1-7	47,296	1,922	6.0 (5.4, 6.4)	1.00	0.95, 1.05	1.06	1.00, 1.11
Relationship satisfaction [c]	1-6	47,161	1,922	5.5 (5.1, 5.8)	1.05	0.97, 1.14	1.08	0.99, 1.18
Use of antidepressants, anxiolytics and/or hypnotics	No	46,836	1,895	NA	Referent		Referent	
	Yes	783	44	NA	1.39	1.04, 1.86	1.24	0.92, 1.67
**Measures of paternal psychosocial stress**								
Symptom Checklist 5 (SCL-5) [b]	1-4	47,133	1,925	1.0 (1.0, 1.2)	1.10	0.95, 1.27	0.99	0.84, 1.17
Satisfaction With Life Scale [c]	1-7	47,191	1,924	5.8 (5.2, 6.2)	0.96	0.92, 1.01	0.98	0.93, 1.03
Relationship Satisfaction [c]	1-6	47,033	1,917	5.4 (5.0, 5.8)	1.03	0.95, 1.11	1.02	0.93, 1.12
Lifetime symptoms of depression [b]	0-5	47,088	1,925	0.0 (0.0, 2.0)	1.05	1.02, 1.09	1.02	0.98, 1.05
Lifetime history of major depressive symptoms	No	42,568	1,746	NA	Referent		Referent	
	Yes	4,520	179	NA	0.97	0.83, 1.12	0.92	0.79, 1.08
Use of antidepressants, anxiolytics and/or hypnotics	No	46,894	1,911	NA	Referent		Referent	
	Yes	725	28	NA	0.95	0.66, 1.37	0.79	0.53, 1.18

CI, confidence interval; NA, not applicable; IQR, interquartile range; RR, relative risk.

[a] Maternal psychosocial stress adjusted for maternal age, parity, education, prepregnancy body mass index, smoking during pregnancy, history of asthma, in addition to the same measure of paternal psychosocial stress. Paternal psychosocial stress adjusted for paternal age, education, body mass index, smoking, history of asthma, in addition to the same measure of maternal psychosocial stress.

[b] Higher scores indicate more stress

[c] Higher scores indicate less stress

Web Table 11. Association between maternal psychosocial stress during pregnancy and offspring asthma at 7 years defined by maternal report through questionnaire, Norwegian Mother and Child Cohort Study, 2000–2007

Measure of Maternal Psychosocial Stress During Pregnancy	Range of Scale	*N*	No. of Cases	Median (IQR)	Unadjusted		Adjusted [a]	
					RR	95% CI	RR	95% CI
Symptom Checklist 5 (SCL-5) at 18 gestational weeks [b]	1-4	36,350	2,004	1.0 (1.0, 1.4)	1.30	1.18, 1.43	1.21	1.09, 1.34
Satisfaction With Life Scale at 18 gestational weeks [c]	1-7	36,586	2,016	6.0 (5.4, 6.4)	0.96	0.92, 0.99	0.99	0.95, 1.03
Relationship Satisfaction at 18 gestational weeks [c]	1-6	35,381	1,940	5.5 (5.0, 5.8)	0.94	0.88, 1.01	0.96	0.90, 1.03
Work stress at 18 gestational weeks [b]	1-4	34,478	1,855	2.0 (1.8, 2.3)	1.24	1.11, 1.38	1.10	0.97, 1.23
Social support at 18 gestational weeks [c]	1-3	35,700	1,960	3.3 (3.0, 3.7)	0.94	0.85, 1.04	1.00	0.90, 1.11
Lifetime symptoms of depression at 18 gestational weeks [b]	0-5	36,584	2,010	1.0 (0.0, 3.0)	1.09	1.06, 1.12	1.07	1.04, 1.10
Lifetime history of major depressive symptoms at 18 gestational weeks	No	28,916	1,495	NA	Referent		Referent	
	Yes	7,668	515	NA	1.30	1.18, 1.43	1.22	1.11, 1.35
Symptom Checklist 5 (SCL-5) at 30 gestational weeks [b]	1-4	36,688	2,017	1.1 (1.0, 1.4)	1.44	1.29, 1.60	1.32	1.18, 1.47
Satisfaction With Life Scale at 30 gestational weeks [c]	1-7	36,743	2,019	6.0 (5.4, 6.4)	0.96	0.92, 1.00	0.99	0.94, 1.03
Relationship satisfaction at 30 gestational weeks [c]	1-6	36,407	2,003	5.5 (5.1, 5.8)	0.96	0.90, 1.03	0.98	0.92, 1.05
Negative life events at 30 gestational weeks [b]	0-8	36,925	2,029	1.0 (0.0, 1.0)	1.18	1.13, 1.22	1.12	1.08, 1.17
Use of antidepressants, anxiolytics and/or hypnotics	No	36,355	1,996	NA	Referent		Referent	
	Yes	570	33	NA	1.05	0.75, 1.47	1.02	0.73, 1.42

CI, confidence interval; IQR, interquartile range; NA, not applicable; RR, relative risk.

[a] Adjusted for maternal age, parity, education, prepregnancy body mass index, smoking during pregnancy and history of asthma.

[b] Higher scores indicate more stress.

[c] Higher scores indicate less stress.

Web Table 12. Association between paternal psychosocial stress and offspring asthma at 7 years defined by maternal report through questionnaire, Norwegian Mother and Child Cohort Study, 2000–2007

Measure of Paternal Psychosocial Stress	Rage of Scale	*N*	No. of Cases	Median (IQR)	Unadjusted		Adjusted [a]	
					RR	95% CI	RR	95% CI
Symptom Checklist 5 (SCL-5) [b]	1-4	28,384	1,552	1.0 (1.0, 1.2)	1.12	0.94, 1.33	1.11	0.94, 1.32
Satisfaction With Life Scale [c]	1-7	28,387	1,552	5.8 (5.2, 6.2)	0.98	0.93, 1.03	0.98	0.93, 1.03
Relationship satisfaction [c]	1-6	28,298	1,552	5.4 (5.0, 5.8)	0.97	0.89, 1.06	0.96	0.88, 1.05
Lifetime symptoms of depression [b]	0-5	28,358	1,553	0.0 (0.0, 2.0)	1.07	1.04, 1.11	1.06	1.03, 1.10
Lifetime history of major depressive symptoms	No	25,755	1,393	NA	Referent		Referent	
	Yes	2,603	160	NA	1.14	0.97, 1.33	1.16	0.98, 1.37
Use of antidepressants, anxiolytics and/or hypnotics	No	28,189	1,536	NA	Referent		Referent	
	Yes	429	429	NA	1.28	0.91, 1.82	1.13	0.77, 1.65

CI, confidence interval; NA, not applicable; IQR, interquartile range; RR, relative risk.
[a] Adjusted for paternal age, education, body mass index, smoking and history of asthma.
[b] Higher scores indicate more stress.
[c] Higher scores indicate less stress.

Web Table 13. Association between maternal psychosocial stress 6 months after delivery and offspring asthma at 7 years defined by maternal report through questionnaire, Norwegian Mother and Child Cohort Study, 2000–2007

Measure of Maternal Psychosocial Stress 6 Months After Delivery	Range of Scale	*N*	No. of Cases	Median (IQR)	Unadjusted		Adjusted Model 1 [a]		Adjusted Model 2 [b]	
					RR	95% CI	RR	95% CI	RR	95% CI
Symptom Checklist 5 (SCL-5) [c]	1-4	36,824	2,022	1.0 (1.0, 1.4)	1.37	1.24, 1.51	1.29	1.15, 1.44	1.22	1.07, 1.39
Satisfaction With Life Scale [d]	1-7	36,562	2,004	6.0 (5.4, 6.4)	0.92	0.88, 0.96	0.95	0.91, 1.00	0.94	0.89, 1.00
Relationship satisfaction [d]	1-6	36,012	1,966	5.4 (5.0, 5.8)	0.92	0.87, 0.98	0.94	0.88, 1.01	0.95	0.87, 1.04
Negative life events [c]	0-10	36,925	2,029	0.0 (0.0, 1.0)	1.23	1.19, 1.28	1.19	1.14, 1.24	1.16	1.11, 1.21
Use of antidepressants, anxiolytics and/or hypnotics	No	36,516	2,007	NA	Referent		Referent		Referent	
	Yes	409	22	NA	0.98	0.65, 1.47	0.84	0.53, 1.33	0.81	0.50, 1.33

CI, confidence interval; NA, not applicable; IQR, interquartile range; RR, relative risk.

[a] Model 1 Adjusted for maternal age, parity, education, smoking during pregnancy, prepregnancy body mass index, smoking the first 6 months of the child's life and history of asthma, in addition to child gender, birth weight, gestational age and breastfeeding the first 6 months.

[b] Model 2 Adjusted the same covariates described in model 1 and the same measure of psychosocial stress during pregnancy.

[c] Higher scores indicates more stress.

[d] Higher scores indicates less stress.

Web Table 14. A sibling analysis of paternal psychosocial stress and offspring asthma at 7 years defined by the Norwegian Prescription Database, Norwegian Mother and Child Cohort Study, 2000–2007

Measure of Paternal Psychosocial Stress	Range of Scale	*N*	No. of Cases	Unadjusted		Adjusted [a]	
				OR	95% CI	OR	95% CI
Symptom Checklist 5 (SCL-5) [b]	1-4	481	237	0.60	0.24, 1.51	0.46	0.16, 1.31
Satisfaction With Life Scale [c]	1-7	483	238	1.22	0.95, 1.57	1.24	0.95, 1.62
Relationship Satisfaction [c]	1-6	479	236	1.33	0.81, 2.20	1.50	0.82, 2.73
Lifetime symptoms of depression [b]	0-5	481	237	1.12	0.90, 1.40	1.16	0.90, 1.49
Lifetime history of major depressive symptoms	No	449	226	Referent		Referent	
	Yes	32	11	0.40	0.15, 1.01	0.46	0.15, 1.36

CI, confidence interval; OR, odds ratio.

[a] Adjusted for paternal age, education, body mass index, smoking and history of asthma.

[b] Higher scores indicate more stress.

[c] Higher scores indicate less stress.
